# Gram-negative resistance and need for ICU among urinary tract infections in the United States

**DOI:** 10.1186/cc12012

**Published:** 2013-03-19

**Authors:** M Zilberberg, A Shorr

**Affiliations:** 1 EviMed Research Group, LLC, Goshen, MA, USA; 2 Washington Hospital Center, Washington, DC, USA

## Introduction

Urinary tract infection (UTI) can lead to both hospitalization and severe sepsis. We theorized that UTI due to Gram-negative (GN) multidrug-resistant *P. aeruginosa *(MDR-PA), extended-spectrum ß-lactamase (ESBL), *E. coli *(EC) and *Klebsiella *sp. (KP), and carbapenemase-producing Enterobacteriaceae (CPE) would be frequently isolated in the ICU.

## Methods

We analyzed a large US-based microbiology database, Eurofins TSN, between the years 2000 and 2009. We determined the proportion of isolates caused by MDR-PA, ESBL-EC, ESBL-KP, and CPE relative to their susceptible counterparts. We defined MDR-PA as any isolate resistant to ≥3 drug classes. ESBL organisms were defined as *E. 3coli *and *K. pneumoniae *resistant to a third-generation cephalosporin. Enterobacteriaceae were considered CPE if resistant to both a third-generation cephalosporin and a carbapenem. We further examined the evolution of the frequency of resistance among GN UTIs over time.

## Results

**Figure 1:**
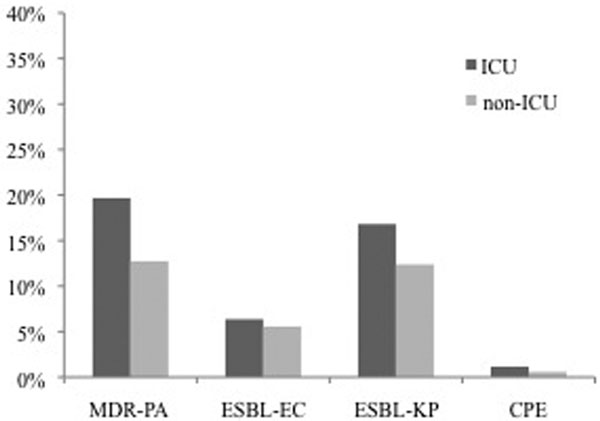


We identified 115,201 PA (13.7% MDR-PA), 359,090 EC (5.6% ESBL), 97,419 KP (12.9% ESBL), and 176,110 Enterobacteriaceae (0.6% CPE) UTI specimens. The prevalence of resistance rose for each organism of interest from 2000 through 2009: MDR-PA, 11.6 to 12.3%; ESBL-EC, 3.3 to 8.0%; ESBL-KP, 9.1 to 18.6%; CPE 0 to 2.3%. For each organism the proportion of resistant pathogens was consistently higher among ICU specimens than among specimens from other hospital locations, reaching nearly 20% for MDR-PA (Figure 1).

## Conclusion

The microbiology of GN UTI hospitalizations has shifted over the last decade and greater antimicrobial resistance is evident. The prevalence of MDR-PA, ESBL-EC, ESBL-KP, and CPE is higher in the ICU than in other hospital locations.

